# 

**Published:** 1906-07-15

**Authors:** 


### CONTENTS.

Event and Comment - - - - - - - - - 325

Review of Pyorrhea Alveolaris Literature, By G. C. Bowles, D.D.S. 329

Oral Hygiene Excerpts, - - - - By N. S. Hoff, D.D.S. 339

Practical Points in Filling, - - - By H. P. Martin, D.D.S. 345

Method for Producing Perfect Joints in Gum-Section Plates, By R. E. Petty, D.D.S. 356

Orthodontia Notes, - - - - - - - W. J. Brady 357

With Our Editors - - - - - - - - - - 359

Indents - - - - - - - - - - - - 368

Announcements - - - - - - - - - - 373

